# Food Should not be Forgotten: Impacts of Combined Cash Transfer Receipt and Food Security on Child Education and Cognition in South Africa and Malawi

**DOI:** 10.1007/s10461-021-03317-6

**Published:** 2021-06-11

**Authors:** Lorraine Sherr, Kathryn J. Roberts, Mark Tomlinson, Sarah Skeen, Helen Mebrahtu, Sarah Gordon, Stefani du Toit, Katharina Haag, Lucie D. Cluver

**Affiliations:** 1 Institute for Global Health, University College London, London, UK; 2 Department of Global Health, Institute for Life Course Health Research, Stellenbosch University, Stellenbosch, South Africa; 3 School of Nursing and Midwifery, Queens University, Belfast, UK; 4 Centre for Evidence-Based Intervention, Department of Social Policy & Intervention, University of Oxford, Oxford, UK; 5 Department of Psychiatry and Mental Health, University of Cape Town, Cape Town, South Africa

**Keywords:** Cash grant, Food security, Sub-saharan africa, Education, Cognition

## Abstract

Social protection can take many forms. Both cash transfers and food security may have important contributions to child cognitive development. This study examines the potential impact of combinations of cash transfers and food security status on child cognitive development and educational outcomes. Cross-sectional data for 796 HIV-affected children in the Child Community Care study were utilised for this analysis. Children and caregivers completed interview schedules comprised of standardised items on socio-demographics, household data, cash grant receipt and food security status, school achievement, and cognition. A series of logistic and linear regression models and marginal effects analyses were undertaken to explore the impacts of differing levels of social protection (none; either cash grant receipt or food secure status or, both in combination) on child educational and cognitive outcomes. Although all children lived in poverty-stricken households, 20% (157/796) of children did not live in a household in receipt of a cash grant and did not report food security; 32.4% (258/796) reported either component of social protection and, 47.9% (381/796) received both measures of social protection in combination. Compared to no social protection, being in receipt of either component of social protection was found to be significantly associated with being in the correct class for age, higher scores of non-verbal cognition, and higher working memory scores. Receiving both social protection measures in combination was found to be significantly associated with reduced educational risk scores, improved odds of being in the correct class for age, regular school attendance, missing less than a week of school in the previous two weeks, higher scores on measures of nonverbal cognition, higher working memory scores, and learning new things more easily. Educational and cognitive outcomes for children can be bolstered by social protection measures (cash grant receipt or food security). Benefits are enhanced when social protection is received in combination. Such findings support the notion of synergistic social protection responses for children living in environments impacted by high levels of HIV burden and deprivation.

## Introduction

Children face many obstacles across the life course. Child survival is contingent on multiple factors including safe birth, avoidance of infections and illnesses, stimulating early environments, access to health care, education, nutrition [1, 2], and good parenting [3, 4]. In sub-Saharan Africa, children carry the burden of many deprivations [5–8]. Social protection is a fundamental priority for children. In HIV-endemic countries, parental illness and child infection has compounded these challenges [1, 5, 9].

Child development is a maturational process. As such—from a life course perspective—the acquisition of skills and opportunities throughout a lifetime are built on the foundational capacities established during early childhood [10]. Child cognitive and educational abilities have lasting implications for not only the individual child as they progress through childhood, adolescence and adulthood (i.e. those individuals who do not reach their developmental potential are projected to lose approximately a quarter of adult average income per year), but also wider society [11]. When a child does not reach their developmental potential during childhood this may be associated with long term negative economic impacts, which in turn, perpetuates cycles of poverty for generations to come. Such negative cycles have widespread impacts for national growth, Gross Domestic Product (GDP) and, global contributions. Sub-Saharan Africa remains the region with the highest number of births [10, 12]. As such, Africa’s child population is expected to reach 1 billion by 2055—making it the largest child population in the world [13]. Successful child development is imperative to ensuring the long-term individual and societal success of this young population.

There is a solid evidence base across numerous geographical settings that cash transfers—both conditional and unconditional—are a potential pathway to ameliorate challenging conditions [14, 15]. Conditional cash transfers—where parents/caregivers are eligible for cash transfers based on meeting certain conditions—which are often child health related and, include items (conditions to be met) such as proven birth certification, immunisation, adherence to antiretroviral therapy and school enrolment [16]. Unconditional transfers have also been introduced with similar benefits [17]. When conditions are not attached, parents/caregivers have still been shown to spend the transfers on health, welfare and educational provision for the children [18]. This obviates the problems associated with conditional cash transfers, where supply of interventions (such as school places, or parental training places) may have been a limiting factor, as well as the dilemma around withholding transfers to those who do not adhere to conditions—but who in fact, may be the most needy. The type of recipient and the circumstances around the transfer are also seen as important factors within the success of cash transfers [19].

There is sound evidence showing the benefits of cash transfer on child outcomes [20]. Much of this evidence emerges from secondary analyses of robust existing data. For example, among adolescents, studies have shown that cash transfers can reduce adolescent risk behaviours and promote adolescent wellbeing [21, 22]. Furthermore, when cash transfers were combined with care in the form of parenting support, the effects were boosted [23]. In some populations where cash transfers alone had no effect, combinations of cash with good parental care were effective. These studies showed that when cash, safe schools and parenting were considered, specific benefits were recorded for adolescent outcomes [24]. Evaluations of purposefully implemented combination programmes (rather than those focusing on secondary analyses) likewise report positive outcomes for adolescents. For example, one study in Tanzania, suggested that cash in combination with financial education, likely reduced female adolescent engagement in transactional sex [25]. Similarly, an evaluation of cash provided in combination with child support services within Zimbabwe was found to reduce adolescent and youth exposure to physical abuse [26]. Despite robust evidence, there has been less evidence of the value of combinations for younger children and younger adolescents. A series of studies of children, utilising secondary data analyses, found that cash plus good parenting was associated with better cognitive development and educational outcomes [27], as well as improved nutrition outcomes [28]. There remains a need to evaluate alternative combinations of provision to gain a better understanding of which approaches are most effective in the bolstering of outcomes for children and younger adolescents.

Food insecurity is widespread in sub-Saharan Africa [29] and cash transfer programmes have often included nutritional components [30]. Nutrition related insufficiencies have been associated with poor child outcomes and even contributed to preventable child deaths [31], highlighting the importance of food security and the potential value of good nutrition within combined social protection [32].

Evaluations of cash transfer programmes to date have focused on a number of outcomes, such as use of health services and health related outcomes [15, 33, 34], HIV infection and sexual risk related to HIV [23, 35, 36], nutrition [37] or education [18]. However, to fully elucidate child development benefit, it is also important to examine the impact of interventions on child cognitive development [38–41]. The majority of studies in this area are concentrated in Central and South America, with fewer insights from Africa [42]. This study is one of the first to evaluate a broad range of child cognitive and educational outcomes within the African context among younger children, where cash grants are part of government provision rather than provided within a research study.

As economic constraints increase, some policymakers are seeking guidance on whether they should abandon some interventions and substitute others—such as moving from nutritional support to cash transfers only. It is therefore important to examine associations of a combined approach where cash transfers are combined with nutritional input to potential booster effects [43]. Both cash transfers and adequate nutrition have important independent contributions to child development. This study examines associations between a combination of cash transfers and food security status on child development outcomes—operationalised as educational outcomes and child cognitive development—to explore potential contributors to child outcomes and the unlocking of child potential within the sub-Saharan African region.

## Methods

### Participants

Data collection was undertaken as part of the Child Community Care study (2013–2014), which tracked psychosocial outcomes of children and families accessing community-based organisations (CBOs) in South Africa and Malawi. Data were drawn from consecutive attenders of 28 CBOs (24 in South Africa, 4 in Malawi). CBOs were randomly selected (stratified by funding organisation and geographical location) from a list of 588 CBOs working within South Africa and/or Malawi drawn from a complete list of all funded programmes from 11 partner organisations (World Vision, UNICEF, Bernard van Leer Association, REPSSI, Stop AIDS Now, the AIDS Alliance, The Diana Memorial Fund, Comic Relief, Help Age, Firelight Foundation and Save the Children). Eight hundred and fifty-four children (5–15 years) and their caregivers were considered for inclusion within the current analyses. Participants were only included if they responded to all measures of interest which resulted in 796 usable cases (93.2%).

### Procedure

Consecutive child attenders (aged 5–15 years) of CBOs and their primary caregivers completed interviews consisting of a battery of standardised and study specific questionnaires inclusive of measures of health, wellbeing, cognition, nutrition, and socio-demographic information. Questionnaires were administered by specifically trained data collectors using mobile phone technology [44]. All participants received detailed information on the study. Informed written consent (caregivers) and assent (children) was also obtained from all participants within the study. Participants completed all questionnaires and consent forms in the language of their choice. All study information, consent forms and questionnaires used existing translations or were translated into Zulu, Xhosa and Chewe, as appropriate, and back translated into English for checking. Ethical approvals for the Child Community Care study were obtained from both University College London (1478/002) and Stellenbosch University (N10/04/112). Within country approvals from CBOs included within the study were also obtained. The analyses presented within this manuscript utilise cross-sectional data.

### Measures

#### Child Characteristics

Socio-demographic information (measured by parental report) was gathered on child biological sex, child age, child HIV status, country of residence and exposure to wealth/poverty. The latter was assessed using an inventory of household assets drawn from the Child Status index tool (CSI; as a proxy indicator). [45, 46] Caregivers indicated which, from a list of ten, material assets (e.g. a bed, access to electricity) a child had access to in their household. Scores ranged from 0 to 10 with a greater number of assets indicative of greater wealth/less poverty [45, 46].

#### Cash Grant Receipt

Grant receipt was determined by caregiver reports. Caregivers were asked whether they received any of the following six available grants; state pension, retirement pension, disability grant, child support grant, foster care grant or care dependency grant or any other cash transfer support. The cash grant measurement used within analyses included both conditional and unconditional grants as well as grants based on specific criteria i.e. child support grants which require recipient household to be below a predetermined income level and for the child to be issued with a birth certificate. A regional approach to analyses taken due to the similarity in programming in countries included within analyses (South Africa and Malawi). Some grants were mutually exclusive. Thus, the final measure of grant receipt was dichotomised recording whether any grant was received versus no grants received (1 = yes/ 0 = no).

#### Food Security Status

For the purpose of analyses, a composite measure of food security status was derived from two sources with reports from both child and primary caregiver reporting items drawn from the CSI tool [45, 46]. Children within the study reported whether they went to bed hungry the previous evening (no; n = 707/yes; n = 89). Caregivers reported whether their child had sufficient food all the time, regularly, less food than needed or regularly had no food to eat). This item was dichotomised to distinguish sufficient food all/most of the time (n = 476) versus not (n = 320). The final composite measure used within analyses was dichotomised to reflect good (child did not go to bed hungry and caregiver reported the household had sufficient food at all/most of the time; n = 446) vs. poor (any other combination of items; n = 350) food security based on both child and caregiver responses to the above-mentioned measures.

#### Educational Outcomes

Questions regarding child educational outcomes were drawn from the CSI tool [45, 46]. Caregivers responded to 5 items relating to child school accessibility and learning outcomes: (1) school attendance (‘Does your child go to school?’ Responses were dichotomised as ‘yes, regularly’ or ‘no, not regularly’); (2) school non-attendance (‘How many days did the child miss school in the past 2 weeks?’ Responses were coded as ‘missed > 1 week’ or ‘missed < 1 week’); (3) being in the correct class for age (‘Is the child in the correct class for his or her age?’ Response ‘yes’ or ‘no’); (4) school performance (‘How do teachers report your child is doing in school?’ Responses were coded as ‘doing as well as or better than most children’ or ‘he or she struggles at school’); (5) learning progression (‘is your child quick to learn when introduced to new chores or things? Response ‘yes’ or ‘no’).

**Number of educational risks** was a composite measure based on the five binary educational outcomes above. Responses were coded to indicate risk i.e. being in the incorrect class, struggling in school, being rated as a slower learner, irregular attendance and missing more than a week of school. Confirmatory responses were coded as 1 for each variable, resulting in a total school ranging from 0–5 with greater scores indicative of greater educational risk.

#### Cognitive Outcomes

**Attention and working memory** were assessed using the digit-span sub-test of the Wechsler Intelligence Scale for Children (WISC-IV) [47]. Children were asked by a trained interviewer to recall a series of dictated digits in series both forwards and backwards. An age-standardised score was recorded (0–20), with higher scores indicative of better attention and working memory.

**Non-verbal cognitive ability** was measured using the draw-a-person task [48]. This screening task is based on children’s ability to draw two human figures. Responses were coded by two researchers independently, using a scoring classification system. Age standardised scoring was recorded for each drawing and mean scores were calculated (range 40–130). Higher scores are indicative of more advanced cognitive ability.

**Cognitive functioning difficulty or disability** was assessed using the Ten Questions screen for childhood disability [49]. Caregivers responded to questions relating to differing domains of child development. Questions relating to **learning**, **remembering**, and **comprehension** (questions were formulated ‘does your child have difficulty…?’ Responses were ‘yes’ or ‘no’) were utilised for the purpose of these analyses.

### Statistical Analyses

All analyses were undertaken using Stata v.15 [50]. Differences between three groups (*1*)* those who reported receiving both a cash grant and being food secure, *(2)* those who reported receiving either a cash grant or being food secure, and *(3)* those who received neither a cash grant nor reported being food secure*) were explored with regard to participant characteristics inclusive of cognitive and educational outcomes using chi-square tests (for categorical outcomes) or ANOVA models (for continuous outcomes). Results are reported using measures of central tendency (mean and standard deviations [SD]) for continuous variables, and frequencies and percentages for categorical variables. Post-hoc tests using Tukey’s-Kramer test for multiple comparisons was used to identify group differences in ANOVA models.

A series of logistic and linear regression models were used to examine the associations of cash grant receipt or being food secure and combined cash grant receipt and being food secure (compared to a reference category reflecting participants neither reporting cash receipt or being food secure) with child cognitive and educational outcomes (reported separately). For all regression analyses presented, *Model 1* shows the unadjusted univariate associations between cash grant receipt and being food secure and, either cognitive or educational outcomes. *Model 2* presents the multivariate associations between exposure variables (cash receipt and good food security status) and the outcome variables (cognitive and educational outcomes) inclusive of potential covariates. *Model 3* uses interaction terms to assess the potential multiplicative effects of cash and nutrition on cognitive or educational outcomes. The exponential coefficients (Beta) of such interactions are presented using linear probability models to aid with interpretation [51, 52]. Where associations between exposure and outcome variables were identified, contrast ratios are presented to further aid in identifying if interactions were additive or synergistic. Covariates identified as being prominent in the existing literature with strong associations (p < 0.2) with both predictor and outcome variables are included in the second and third step models. This includes child gender, child age, child disability, child HIV status, child school status and number of household assets. Unadjusted and adjusted odds ratios (ORs and aORs, respectively) with 95% confidence intervals (95% CI) are reported.

Marginal effects analyses were undertaken to further explore the impact of cash grant receipt and food security status on child educational and cognitive outcomes where associations were identified. Probability predictions for binary outcomes, adjusted for covariates, with 95% confidence intervals are presented.

## Results

### Participant Characteristics

Most children in the sample (82.0%; 653/796) lived in South Africa and 18.0% (143/796) in Malawi. Just over half (52.3%; 416/796) of the children were female. The average age of children in the sample was 10.5 years (SD: 2.61), while 13.8% (110/796) of the children were reported to be living with HIV. The sample on the whole high poverty levels, with an average number of reported household assets of 3.88 (SD: 1.94; Range 0–10).

### Household Cash Grant Receipt and Food Security Status

Two-thirds (66.4%; 574/796) of the children lived in a household in receipt of a cash grant. Over half (56.0%; 446/796) reported food security (based on both child and caregiver report). Overall, 19.7% (157/796) did not live in a household in receipt of a cash grant and did not report food security (child and caregiver report). Just under a third (32.5%; 258/796) lived in a household in receipt of a cash grant or reported food security, and 47.9% (381/796) reported living in a household in receipt of a cash grant and being food secure. Comparative to South Africa (15.9%), a higher proportion of children in Malawi (84.1%) were living in a household that did not receive a cash grant and was classified as being food insecure. Children living in households in receipt of a cash grant and that was food secure were found to report having access to a greater number of household assets comparative to those receiving either cash or being food secure, or no component of social protection. A trend for group differences relating to HIV status was identified; a greater proportion of children living with HIV were identified in the group classified as receiving no cash grant and being food insecure (19.9%) compared to those classified as receiving a cash grant and/or being food secure (12.6%/12.0%; see Table 1).

**Table 1:** Sample outcomes stratified by cash and food security status (n = 796)

	Total (n = 796)	Cash plus food security(n = 381)	Cash or food secure (n = 258)	No cash, food insecure (n = 157)	*F/X*^2^, p value
Socio-demographics					
*Country*					581.91, 0.000
South Africa	653 (82.0%)	381 (100%)	247 (95.7%)	25 (15.9%)	
Malawi	143 (18.0%)	0 (0.00%)	11 (4.30%)	132 (84.1%)	
Child biological sex (female)	416 (52.3%)	205 (53.8%)	132 (51.2%)	79 (50.3%)	0.73, 0.70
Child Age (years)	M: 10.48 (SD: 2.61)	M: 10.22 (SD: 2.62)^a^	M: 10.39 (SD: 2.60)^a^	M: 11.30 (SD: 2.46)	0.89, 0.64
Child living with HIV	110 (13.8%)	48 (12.6%)	31 (12.0%)	31 (19.9%)	5.97, 0.05
No. Household assets (0–10)	M: 3.88 (SD: 1.94)	M: 4.48 (SD: 1.67)^a^	M: 4.30 (SD: 1.69)^a^	M: 1.75 (SD: 1.40)	7.76, 0.02
Educational outcomes					
Number of educational risks (0–5)	M: 0.85 (SD:1.15)	M: 0.65 (SD: 0.95)^a^	M: 0.84 (SD: 1.11)^a^	M: 1.36 (SD: 1.27)	19.93, < 0.0001
Correct class for age	521 (66.3%)	284 (74.7%)	185 (72.8%)	52 (34.2%)	86.99, < 0.0001
Regular attendance	747 (95.0%)	375 (98.7%)	241 (94.9%)	131 (86.2%)	35.9, < 0.0001
Quick leaner	560 (72.2%)	282 (75.6%)	170 (67.5%)	108 (71.5%)	5.00, 0.08
Doing as well as or better than most in school	653 (83.1%)	326 (85.8%)	207 (81.5%)	120 (79.0%)	4.28, 0.12
Missed less than a week of school	772 (98.2%)	379 (99.7%)	253 (99.6%)	140 (92.1%)	40.27, < 0.0001
Cognitive outcomes					
Draw-a-person score (40–130)	M: 91.45 (SD: 17.19)	M: 96.05 (SD: 14.28)	M: 93.78 (SD: 15.85)	M: 76.25 (SD: 17.47)	9.78, 0.008
Digit span score (0–20)	M: 8.84 (SD: 3.55)	M: 9.14 (SD: 3.34)	M: 9.30 (SD: 3.76)	M: 7.34 (SD: 3.29)	5.17, 0.08
No cognitive functioning difficulty—Learning	664 (83.4%)	344 (90.3%)	215 (83.3%)	105 (66.9%)	44.05, < 0.0001
No cognitive functioning difficulty—Remembering	568 (71.4%)	281 (73.8%)	192 (74.4%)	95 (60.5%)	11.29, 0.004
No cognitive functioning difficulty— Comprehension	766 (96.2%)	369 (96.9%)	248 (96.1%)	149 (94.9%)	1.17, 0.56

Tukey’s post hoc test undertaken to identify mean differences between groups (for continuous variables only)

^a^Statistically different from the No *cash grant receipt, food insecure* group (p =  < 0.05)

Children in households with no receipt of cash grant and classified as being food insecure were identified as having a greater number of educational risks, were less likely to be in the correct class for age, were less likely to attend school regularly and, less likely to have missed less than a week of school in the previous two weeks comparative to those children in receipt of a cash grant and/or being food secure. Similarly, in the assessment of cognitive outcomes these children were identified as having lower average scores in the assessment of non-verbal cognitive ability, and caregivers were more likely to report difficulties in learning and remembering new things compared to those receiving cash grants and/or being food secure (see Table 1).

### Associations Between Household Cash Grant Receipt/Food Security and Child Education/Cognitive Outcomes

Children living in a household in receipt of *either* component of interest (cash grant, good food security status) had greater odds of being in the correct class for age comparative to those children who received neither component of interest. Being food secure *and* receiving a cash grant were associated with reduced educational risk scores, greater odds of being in the correct class for age, greater odds of regular school attendance and greater odds of missing less than a week of school in the previous two weeks (see Table 2).

**Table 2:** Cross-sectional regression models exploring associations between cash grant receipt and good nutrition, and child educational outcomes (n = 796)

	Total no. educational risks (0–5)	Correct class for age	Regular attendance	Quick learner	Doing as well as or better than most in school	Missed less than a week of school
	B (95% CI)	OR (95% CI)	OR (95% CI)	OR (95% CI)	OR (95% CI)	OR (95% CI)
Model 1						
No cash, food insecure (n = 157)	1 (Ref)	1 (Ref)	1 (Ref)	1 (Ref)	1 (Ref)	1 (Ref)
Either cash or food security (n = 258)	− 0.53 (− 0.74, − 0.31)***	5.16 (3.34–7.96)***	2.97 (1.44–6.12)**	0.83 (0.53–1.28)	1.17 (0.71–1.94)	21.69 (2.79–168.52)**
Cash plus food security (n = 381)	− 0.71 (− 0.92, − 0.51)***	5.68 (3.79–8.55)***	12.02 (4.44–32.53)***	1.23 (0.81–1.88)	1.61 (0.99–2.61)	32.49 (4.18–252.1)**
Model 2						
No cash, food insecure (n = 157)	1 (Ref)	1 (Ref)	1 (Ref)	1 (Ref)	1 (Ref)	1 (Ref)
Either cash or food security (n = 258)	− 0.22 (− 0.46, 0.02)	2.29 (1.33–3.92)**	1.69 (0.68–4.21)	0.73 (0.43–1.24)	0.71 (0.38–1.32)	5.81 (0.60–55.89)
Cash plus food security (n = 381)	− 0.38 (− 0.61, − 0.15)**	2.36 (1.39–4.00)**	6.49 (2.03–20.7)**	1.10 (0.65–1.85)	0.99 (0.53–1.85)	9.59 (1.01–90.34)*
Model 3						
No cash, food insecure (n = 157)	1 (Ref)	1 (Ref)	1 (Ref)	1 (Ref)	1 (Ref)	1 (Ref)
Cash	− 0.14 (− 0.39, 0.11)	2.13 (1.21–3.75)**	1.43 (0.54–3.77)	0.61 (0.35–1.04)	0.60 (0.32–1.15)	3.76 (0.36–39.15)
Food security	− 0.46 (− 0.79, − 0.14)**	2.86 (1.31–6.26)**	2.95 (0.61–14.22)	1.55 (0.70–3.43)	1.29 (0.50–3.38)	2.46 (0.14–41.41)
Interaction—Cash BY Food security^a^	0.22 (− 0.15, 0.59)	− 0.21 (− 0.36, − 0.06)***	− 0.04 (− 0.12, − 0.03)	0.05 (− 0.11, 0.21)	0.03 (− 0.10, 0.16)	− 0.07 (− 0.11, − 0.02)**

**Model 1:** Univariate regression analyses showing associations of cash or food security and combined cash and food security with child educational outcomes

**Model 2:** Multivariable regression analyses showing associations of cash or food security and combined cash and food security with child educational outcomes controlling for covariates: child biological sex (female), child age (years), child HIV status (positive), number of household assets (proxy wealth indicator)

**Model 3:** Multivariable regression analyses showing the interaction between cash grant receipt and food security, and child educational outcomes controlling for covariates: child biological sex (female), child age (years), child HIV status (positive), number of household assets (proxy wealth indicator)

*B* Beta, *OR* Odds Ratio, *CI* confidence interval

p < 0.05, *p < 0 .01, **p ≤ 0.001, ***

^a^Linear probability models denoted by *Beta (95% confidence intervals)*

Receiving *either* component of interest was also found to be associated with improved outcomes for two cognitive measures: non-verbal cognitive ability and, attention and working memory. Receiving a *combination of both* a household grant and being food secure was found to be associated with three cognitive measures: nonverbal cognitive ability, attention and working memory and not having any difficulty learning new things (see Table 3).

**Table 3:** Cross-sectional logistic regression models exploring associations between cash grant receipt and food security status, and child cognitive outcomes (n = 796)

	Performance on cognitive tests		No cognitive functioning difficulty or disability		
	Draw-a-person (40–130)	Digit span (0–20)	Learning	Remembering	Comprehension
	B (95% CI)	B (95% CI)	OR (95% CI)	OR (95% CI)	OR (95% CI)
Model 1					
No cash, food insecure (n = 157)	1 (Ref)	1 (Ref)	1 (Ref)	1 (Ref)	1 (Ref)
Either cash or food security (n = 258)	17.52 (14.43,20.61)***	1.96 (1.27–2.70)***	2.48 (1.55–3.94)***	1.89 (1.24–2.90)**	1.33 (0.51–3.45)
Cash plus food security	19.79 (16.89,22.69)***	1.80 (1.15–2.45)***	4.60 (2.86–7.40)***	1.83 (1.24–2.72)**	1.65 (0.66–4.12)
Model 2					
No cash, food insecure (n = 157)	1 (Ref)	1 (Ref)	1 (Ref)	1 (Ref)	1 (Ref)
Either cash or food security (n = 258)	15.15 (11.60,18.70)***	1.22 (0.43, 2.01)**	1.72 (0.97–3.04)	1.55 (0.94–2.56)	0.50 (0.17–1.53)
Cash plus food security (n = 381)	17.46 (14.00,20.92)***	1.02 (0.25, 1.79)**	3.13 (1.74–5.61)***	1.48 (0.91–2.41)	0.59 (0.20–1.75)
Model 3					
No cash, food insecure (n = 157)	1 (Ref)	1 (Ref)	1 (Ref)	1 (Ref)	1 (Ref)
Cash	16.05 (12.37, 19.74)***	1.27 (0.44, 2.09)**	1.79 (0.98–3.28)	1.51 (0.90–2.55)	0.52 (0.15–1.73)
Food security	12.22 (7.38, 17.06)***	1.07 (− 0.02, 2.15)	1.55 (0.70–3.40)	1.68 (0.83–3.40)	0.48 (0.11–2.07)
Interaction cash BY food security^a^	− 10.89 (− 16.34, − 5.44)***	− 1.32 (− 2.54, − 0.10)*	− 0.04 (− 0.17, 0.09)	− 0.12 (− 0.28. 0.04)	0.03 (− 0.04, 0.10)

**Model 1:** Univariate regression analyses showing associations of cash or food security and combined cash and food security with child cognitive outcomes

**Model 2:** Multivariable regression analyses showing associations of cash or food security and combined cash and food security with child cognitive outcomes controlling for covariates: child biological sex (female), child age (years), child HIV status (positive), number of household assets (proxy wealth indicator)

**Model 3:** Multivariable regression analyses showing the interaction between cash grant receipt and food security, and child cognitive outcomes controlling for covariates: child biological sex (female), child age (years), child HIV status (positive), number of household assets (proxy wealth indicator)

*B* Beta, *OR* Odds Ratio, *CI* confidence interval

p < 0.05, *p < 0 .01, **p ≤ 0.001, ***

^a^Linear probability models denoted by *Beta (95% confidence intervals)*

For most outcomes found to be associated with combined cash grant receipt and food security, no statistically significant interactions between cash grant receipt and food security status were apparent, indicative of no synergistic effects (model 3; Tables 2 and 3). However, synergistic effects were identified relating to children being in the correct class for age (F = 7.53, p = 0.006), missing less than a week of school (F = 7.47, p = 0.006; see Education [Table 2]), scores on the draw-a-person assessment of non-verbal cognitive ability (F = 15.37, p = 0.0001) and, scores on the digit span test (F = 4.50, p = 0.03; see Cognition [Table 3]).These synergistic effects indicate that the joint associations of receiving a cash grant and being food secure in combination differs from the independent effects of receiving either intervention alone. For these outcomes, the effect of social protections in the form of cash grant receipt and food security in combination is larger than the independent effects of each provision on the outcomes of interest. Antagonistic interactions were identified in relation to educational risk scores (F = 1.37, p = 0.24; see Education [Table 2]), regular attendance at school (F = 1.20, p = 0.27) and no difficulty learning new things (F = 0.43, p = 0.51; see Cognition [Table 3]).

**Fig. 1:**
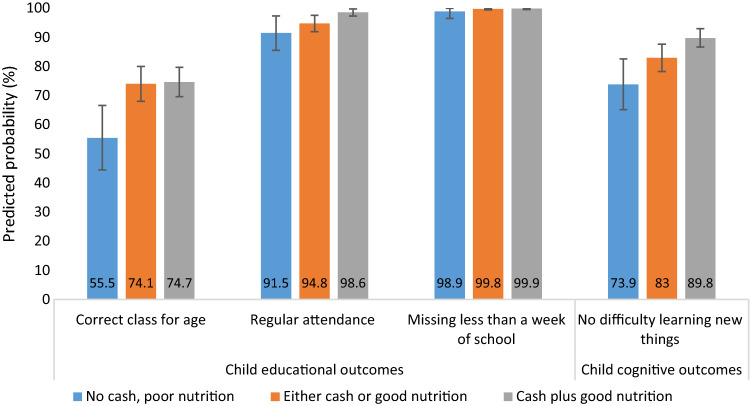
Probability predictions ascertained from marginal effects models testing exploring the effects of cash and food security on child educational and cognitive outcomes (binary). Adjusted for child biological sex (female), child age (years), child HIV status (positive), number of household assets (proxy wealth indicator)

To explore potential additive associations of cash grant receipt and food security amongst outcomes further, estimates of the predicted probability of educational and nutritional outcomes were calculated for binary outcomes, controlling for all predictor variables (see Fig. 1). Predicted probability of regular school attendance was 91.5%% when no cash grant was received or food security was reported, 94.8% when *either* a cash grant was received, or food security was reported and 98.6% when both a cash grant was received and food security was reported in combination. Similar patterns were identified regarding being in the correct class for age (55.5%, 74.1% and 74.7%, respectively) and not having any difficulty learning new things (73.9%, 83.0% and 89.8%, respectively). Most children within the sample did not miss less than a week of school within the previous two weeks, thus predicted probabilities were found to be similar (98.9%, 99.8% and 99.9%, respectively for the three categories of interest; see Fig. 1). Tables 2 and 3 show the additive associations of for continuous variables found to be associated with cash grant receipt and good food security status (educational risk score, non-verbal cognitive ability and, attention and working memory). These models show the discrete change in scores relating to receiving either intervention or both interventions in combination from the base level (no cash grant and food insecurity).

## Discussion

In this study we show that receiving either a cash grant or being food secure had positive impacts for both educational outcomes (being in the correct class for age) and cognitive outcomes (non-verbal cognitive ability, attention and working memory) among children affected by HIV. This was further enhanced when children received both components in combination, with children demonstrating improved outcomes on educational outcomes, and cognitive outcomes, non-verbal cognition scores and being in the correct class for age.

It is important also to note that for some outcomes, these components had no effect. These data suggest that combination interventions may be more effective for improving outcomes across a range of cognitive and educational indicators for children but are not a catch all solution and other types of intervention combinations may need to be explored. For some of the cognitive outcomes, a lack of impact remains unsurprising as difficulties i.e. with memory and comprehension may indicative of broader, more a complex developmental delay that is unlikely to be widely impacted by the components of interest within this study. For those children living with HIV or HIV exposed uninfected children in the sample, such cognitive difficulties may be a result of disease progression and structural alterations within the brain [53, 54]. Among children experiencing developmental delays, alternative intervention may be required inclusive of developmental screening, surveillance, and tailored interventions to improve developmental outcomes e.g. home stimulation programmes and parental/caregiver support (both of which have been found to be effective among children living with HIV, exposed to HIV [55, 56]. Results from these analyses do also suggest a need for integrated planning and processing. By combining interventions and looking at a broader range of child outcomes, these analyses contribute to dialogue exploring a shift away from siloed approaches to intervention and explores pathways for enhanced impact and harmony of public provision. These findings endorse the importance of a more rounded approach to families that allows for strategic approaches for those who carry very high levels of risk and adversity in multiple domains.

The findings are consistent with previous studies in which combined approaches to social protection (i.e. cash plus care) were found to have beneficial effects for child and adolescent outcomes compared to single intervention provision [14, 42] and, highlight the potential beneficial role of attaining food security within combined approaches. Child malnutrition remains a prominent issue within sub-Saharan Africa [29, 57], with the current study highlighting that there would be clear benefits to addressing this. The attainment of food security can be achieved through numerous avenues inclusive of providing direct nutritional support, household assets, and improving access to food sources [32]. There is strong evidence that cash transfers themselves improve household food consumption [32, 58] and, as such, cash interventions alone may have an impact. However, as these analyses detail, combination approaches may have bolstering effects and thus, interventions ensuring direct food provision may be necessary to bolster outcomes. The way forward needs to incorporate a strategic approach where the benefits of different intervention combinations is understood, and gaps are highlighted. In this study, all children, by definition, were vulnerable—living in HIV infected or affected families. The needs of HIV exposed and uninfected children have emerged [59], and these children will represent future groups as treatment impacts to reduce mother to child transmission [60]. Despite the availability of grants, many children did not receive them. Children with HIV themselves should have heightened eligibility for grants, and our findings suggest they were significantly less likely to receive grants. Reaching the hard-to-reach children and enabling broad access to interventions remains a challenge.

In this study context, children affected by HIV (living with HIV, living within HIV-affected household, living within high HIV-endemic countries or being HIV-exposed uninfected) face additional burdens (i.e. through virus exposure and social determinants such as poverty). As such, these children may be disadvantaged with regard to access and engagement with education [61], and such burdens, both biological and social, may have negative implications for cognition [62]. These data suggest that engagement with education and some cognitive outcomes for such children may be enhanced through combining social protection interventions that are often already in place within many low and middle income communities.

The sustainable development goals provide a challenge for governments to work towards optimal child development. Multiple services and accelerated impacts [24] may provide a strategic pathway to achieve this. The constant call for novel approaches may instead be substituted with a syndemic approach where well-established existing interventions can be harnessed in a coordinated way to provide multiple avenues of support. This data provides a blueprint for such strategies, which may be needed in the light of global pandemic driven changes (such as the current Covid-19 response) where demand is enhanced, and resources become scarce.

### Strengths and Limitations

This study utilises a large sample of children identified through CBOs living in two high HIV endemic countries within sub-Saharan Africa (South Africa and Malawi) to explore the impact of cash grant receipt and good nutritional status on a broad range of child educational and cognitive outcomes. This unique community level data provides insight into the impact of social protection on child outcomes. However, there were several limitations worth considering. Firstly, these data are cross-sectional, drawn from a study with a non-randomised study design which allows for less certainty regarding causality. Secondly, the exposure variables utilised are based on self-report measures which may bias some of the findings reported. However, these analyses report on field data from a particularly hard to reach population and similar self-report measures have been utilised in previous studies [27, 28, 42]. As such, the large sample reporting on such measures within a resource confined setting provides a novel and unique contribution to the literature regarding combined social protection for this population. Thirdly, in line with previous studies in the field [23, 26, 27, 42], theses analyses focused on the mechanism of cash transfers as a form of social protection, and it was beyond the scope of this study explore cash grants according to reason for receipt i.e. pension/child support grant. Despite similar policy and programming development between South African and Malawi, the distribution of cash grants varied between countries, with receipt substantially lower in Malawi; possibly due to variation within the conditionality of cash grants and the roll out of national programming. This study was set up to report on regional data and as such specific country level outcomes are not reported. Future research directive may benefit from exploring whether the impact of cash transfers differs according to mode of and reason for receipt, as well as specific outcomes stratified by country. Fourthly, due to the small number of participants experiencing some of the outcome variables (i.e. missing less than a week than school), these variables are not as robust and thus findings require caution within interpretation. Despite the small sample size, there is still movement within the observed difference between groups, indicating that social protection may have positive impacts for those more vulnerable groups i.e. those children not attending school on a regular basis. Fifthly, given all participants were accessing CBO support, the potential impact of selection bias on the outcomes explored should also remain a consideration. However, it should be noted that these children have previously been identified as being particularly vulnerable [63]. While it was beyond the scope of these analyses to explore the potential impact of the services provided CBOs, such analyses may benefit future research. Sixthly, to ensure a robust sample size, this study focuses on children and adolescents (5–15 years), future research directive may benefit from stratifying finding by age further identify whether the impacts of social protection differ for younger/older children. Finally, these data also do not report on a specific intervention programmes and, rely on child and caregiver report of cash or nutrition receipt. However, it should be noted that the use of both caregiver and child report relating to food security status aids in the robustness of the measure.

## Conclusions

These data indicate that educational and cognitive outcomes for children affected by HIV can be bolstered by social protection measures (cash grant receipt and food security), particularly when delivered in combination. Such findings support previous literature advocating for synergistic social protection responses for both children and adolescents living in environments impact by high levels of HIV burden and deprivation. Findings also support calls for complex programming models to enhance the efficacy of interventions to promote positive child development.
